# A novel method of ultrasound-guided positive staining using indocyanine green fluorescence in laparoscopic anatomical liver resection of segments VII and VIII

**DOI:** 10.3389/fonc.2023.1138068

**Published:** 2023-02-20

**Authors:** Zedong Jiang, Bo Zhou, Xiang Zheng, Guogang Li, Zhenzhen Gao, Yang Tian, Chunlong Shao, Shaoyan Xu, Sheng Yan

**Affiliations:** ^1^Department of Hepatobiliary and Pancreatic Surgery, The Second Affiliated Hospital Zhejiang University School of Medicine, Hangzhou, China; ^2^Key Laboratory of Precision Diagnosis and Treatment for Hepatobiliary and Pancreatic Tumor of Zhejiang Province, The Second Affiliated Hospital Zhejiang University School of Medicine, Hangzhou, China

**Keywords:** laparoscopic anatomical liver resection, right superior segments, ICG-positive staining, novel method, three-dimensional (3D) simulation, laparoscopic ultrasound, fluorescence imaging

## Abstract

**Background:**

Recently, in many Asian centers, laparoscopic anatomical liver resection (LALR) using the indocyanine green (ICG) fluorescence imaging technique has been increasingly applied in resecting hepatocellular carcinoma, even in colorectal liver metastases. However, LALR techniques have not been fully standardized, especially in right superior segments. Due to the anatomical position, prevailing positive staining using a PTCD (percutaneous transhepatic cholangial drainage) needle was superior to negative staining in right superior segments hepatectomy, while it was difficult to manipulate. Herein, we design a novel method of ICG-positive staining for LALR of right superior segments.

**Methods:**

Between April 2021 and October 2022, we retrospectively studied patients in our institute who underwent LALR of right superior segments using a novel method of ICG-positive staining, which comprised a customized puncture needle and an adaptor. Compared to the PTCD needle, the customized needle was not limited by the abdominal wall and could be punctured from the liver dorsal surface, which was more flexible to manipulate. The adapter was attached to the guide hole of the laparoscopic ultrasound (LUS) probe to ensure the precise puncture path of the needle. Guided by preoperative three-dimensional (3D) simulation and intraoperative laparoscopic ultrasound imaging, we punctured the transhepatic needle into the target portal vein through the adaptor and then slowly injected 5-10 ml of 0.025 mg/ml ICG solution into the vessel. LALR can be guided by the demarcation line under fluorescence imaging after injection. Demographic, procedural and postoperative data were collected and analyzed.

**Results:**

In this study, 21 patients underwent LALR of the right superior segments with ICG fluorescence-positive staining, and the procedures had a success rate of 71.4%. The average staining time was 13.0 ± 6.4 min, the operative time was 230.4 ± 71.7 min, R0 resection was 100%, the postoperative hospital stay was 7.1 ± 2.4 days, and no severe puncture complications occurred.

**Conclusions:**

The novel customized puncture needle approach seems to be feasible and safe for ICG-positive staining in LALR of right superior segments, with a high success rate and a short staining time.

## Introduction

It is well established that for HCC (hepatocellular carcinoma), anatomical liver resection is associated with better overall survival and a lower recurrence rate, and recent evidence suggests its role in the treatment of intrahepatic cholangiocarcinoma and potentially for CRLM (colorectal liver metastases) (1).

Combined with the rapid advance of minimally invasive surgery, laparoscopic anatomic liver resection (LALR) has recently become the preferred procedure in liver resection. Segmentectomy or subsegmentectomy, typically involving resection of the tumor-bearing portal pedicles, without disrupting the structural integrity of remnant segments, is considered LALR (2, 3). It is commonly guided by a demarcation line after portal staining or inflow clamping of the target territory (4), injecting the chemical dye into the portal vein of the tumor-bearing segment by ultrasound-guided transhepatic puncture (5), or inducing the ischemic line by clamping the portal pedicle supplying the segment(s) (6).

The right superior segments, including hepatic segments VII (S7) and VIII (S8), are located deep and adjacent to the hepatic veins and inferior vena cava, making it difficult to use hepatic staining and preoperative three-dimensional simulation guidance during laparoscopy (6). Due to the poor anatomical position, right superior segment resection is considered the most difficult in LALR. There is no standardized approach to right superior segments, although many authors have proposed new and different technical approaches, such as the hilar Glissonean-First approach, transparenchymal approach and hepatic vein first approach (7).

Indocyanine green (ICG) was so common among liver surgeons as a reagent for estimating hepatic function that they could hardly think of the other application of ICG for fluorescence imaging (8). With a fusion monitor mounted on the laparoscopic system, the ICG fluorescence imaging technique offers a novel navigation tool to guide liver parenchymal transection by precisely tracing its segmental territorial borderline (9). This technique was first reported by Prof. Aoki (10) in 2008 and then applied to laparoscopic hepatectomy under the name of positive staining (portal staining) and negative staining(systematic staining after target Glissonean pedicle clamping) technique described by Ishizawa et al. (11) in 2012. With the development of the technique, current consensus guidelines for its use (12) were developed in 2021, and an assessment including 51 eligible reports indicated a satisfactory success rate in terms of delineating targeted hepatic segments (13).

However, LALR techniques have not been fully standardized because both selective inflow portal clamping and conventional portal staining, when used, are difficult to perform in the minimally invasive setting (1), especially in right superior segments. Negative staining is technically demanding and has a potential risk for biliary injury during the Glissonean approach in laparoscopic segment VII and VIII segmentectomy (14). Ultrasound-guided positive staining may be superior to negative staining in right superior segments hepatectomy, while the prevailing positive staining using PTCD needles is difficult to manipulate and hard to learn. Herein, we describe a novel method of positive staining in right superior segments hepatectomy to better achieve the staining goal and explore its feasibility, success rate, efficiency and safety.

## Materials and methods

### Patients

Between April 2021 and October 2022, patients who underwent LALR at the Second Affiliated Hospital of Zhejiang University were studied retrospectively. The inclusion criteria of this study: diagnosed as HCC or CRLM confined to S7 or S8; resectable by conventional laparoscopic surgery; mono-segmentectomy or sub-segmentectomy of right superior segments, including S7, S8, S8v (ventral segment of S8) or S8d (dorsal segment of S8) resection; and the operations were performed by our novel ICG-positive staining. Patients who received ICG-positive staining for LALR but finally received non-LALR (local hepatectomy or conversion to laparotomy), combined with other organ resections or received segments 2 to 6 resections were excluded from the study. Patients underwent a liver functional reserve test based on the plasma ICG retention rate at 15 min 5-7 days before surgery (0.5 mg/kg) (9). Patients with ICG allergy were prohibited from undergoing this procedure. All operations were performed by the same surgical team. Collection data included demographic data (sex, age, pathology, segments of staining, tumor sizes, hepatitis, cirrhosis), procedural data (staining time, staining success rate, operative time, blood loss, R0 resection) and postoperative data (puncture complication, postoperative hospital days). All patients provided written informed consent for surgery, and this study was approved by the Ethical Review Board of the Second Affiliated Hospital of Zhejiang University.

### Preoperative 3D simulation

Preoperative 3D simulation (Vitaworks, Huishi Medical Technology, Shanghai, China) of liver computed tomography (CT) was performed to obtain preliminary knowledge of the location and size of the tumor(s), intrahepatic vascular anatomy and their spatial relationships to the tumor(s). During this preoperative simulation, the number and size of target portal branches, the exact injection point(s), and the resultant positive staining segmental volume were determined (14).

### ICG fluorescence imaging

The fluorescence imaging system (HyPixel™ R1, Mindray, Shenzhen, China) can be used with indocyanine green to provide real-time visible light images and near-infrared fluorescence images. After ICG staining, the fluorescent mode helps surgeons determine the superficial and intrahepatic boundaries according to the fluorescence demarcation.

### Customized puncture needle, adaptor and laparoscopic ultrasound (Pro Focus 2202, BK Medical, Herlev, Denmark)

**Figure 1:**
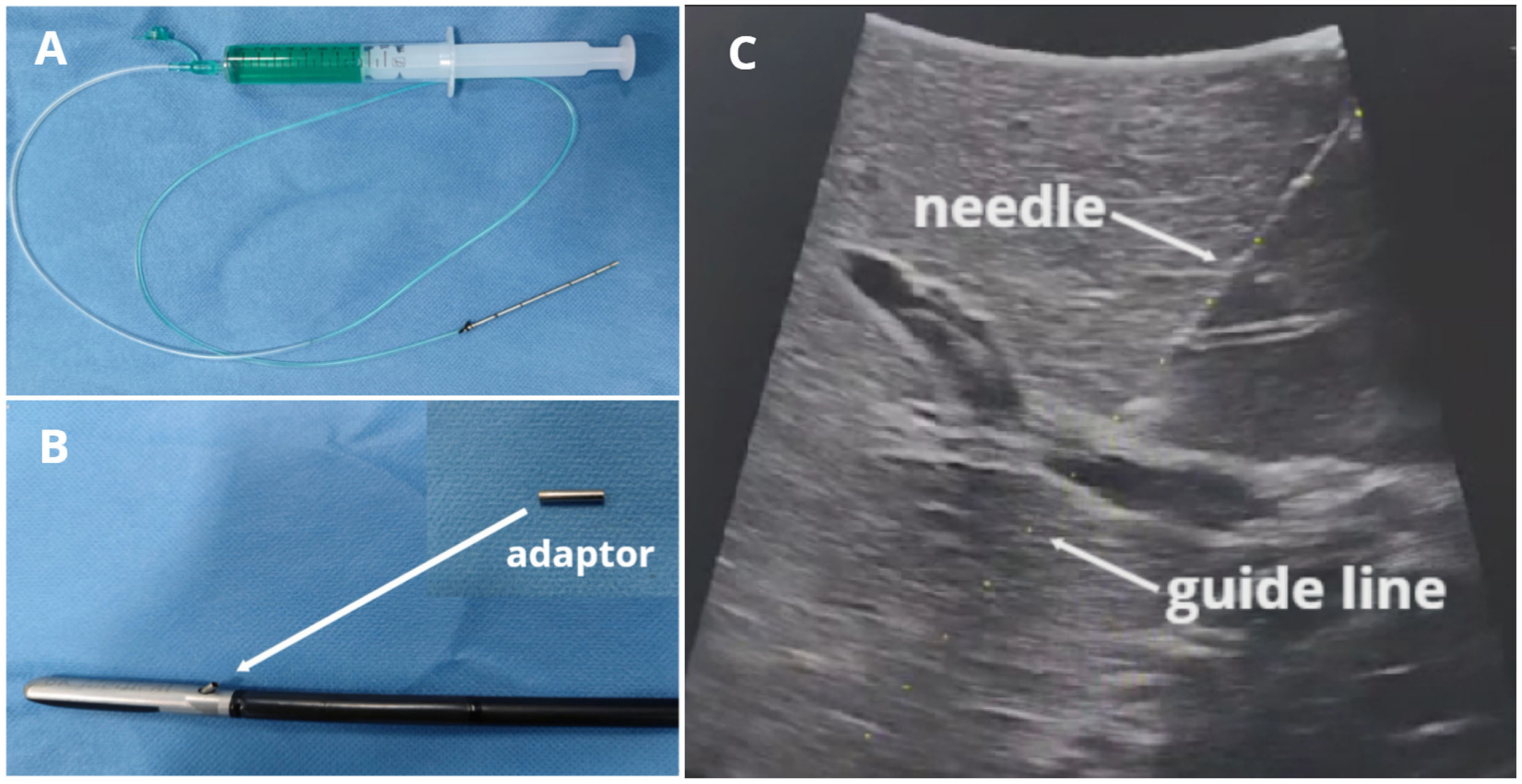
BK LUS-guided precise puncture with our customized needle and adaptor. **(A)** Puncture needle; **(B)** Laparoscopic ultrasound probe and adaptor; **(C)** Puncture needle and the guide line with the same path.

The customized puncture needle is simply connected by a 16-gauge needle (diameter 1.69 mm, length 7 cm) and a soft injection tube (diameter 1.84 mm, length 60 cm) (**Figure 1A**). The BK laparoscopic ultrasound probe is equipped with a puncture hole that guides the puncture. Accordingly, there is a guide line (switched on if we need it) on ultrasound imaging that matches the path of the puncture guide hole. But the guide hole(13-gauge) is larger than the 16-gauge needle. Therefore, we designed an adaptor (a metal sheath) that can precisely match the 16-gauge needle and the 13-gauge guide hole (**Figure 1B**) so that it can guide the needle into the target portal vein precisely according to the guide line (**Figure 1C**). The adaptor was created by an engineer according to our design. It is reusable by high-pressure steam sterilization and can be 3D printed. After our constant practice, we attached the adaptor to the ultrasound hole with a silk thread before the LUS probe entered the abdominal cavity. It was convenient and effective, which could prevent the adaptor from missing.

### Positive staining method

**Figure 2:**
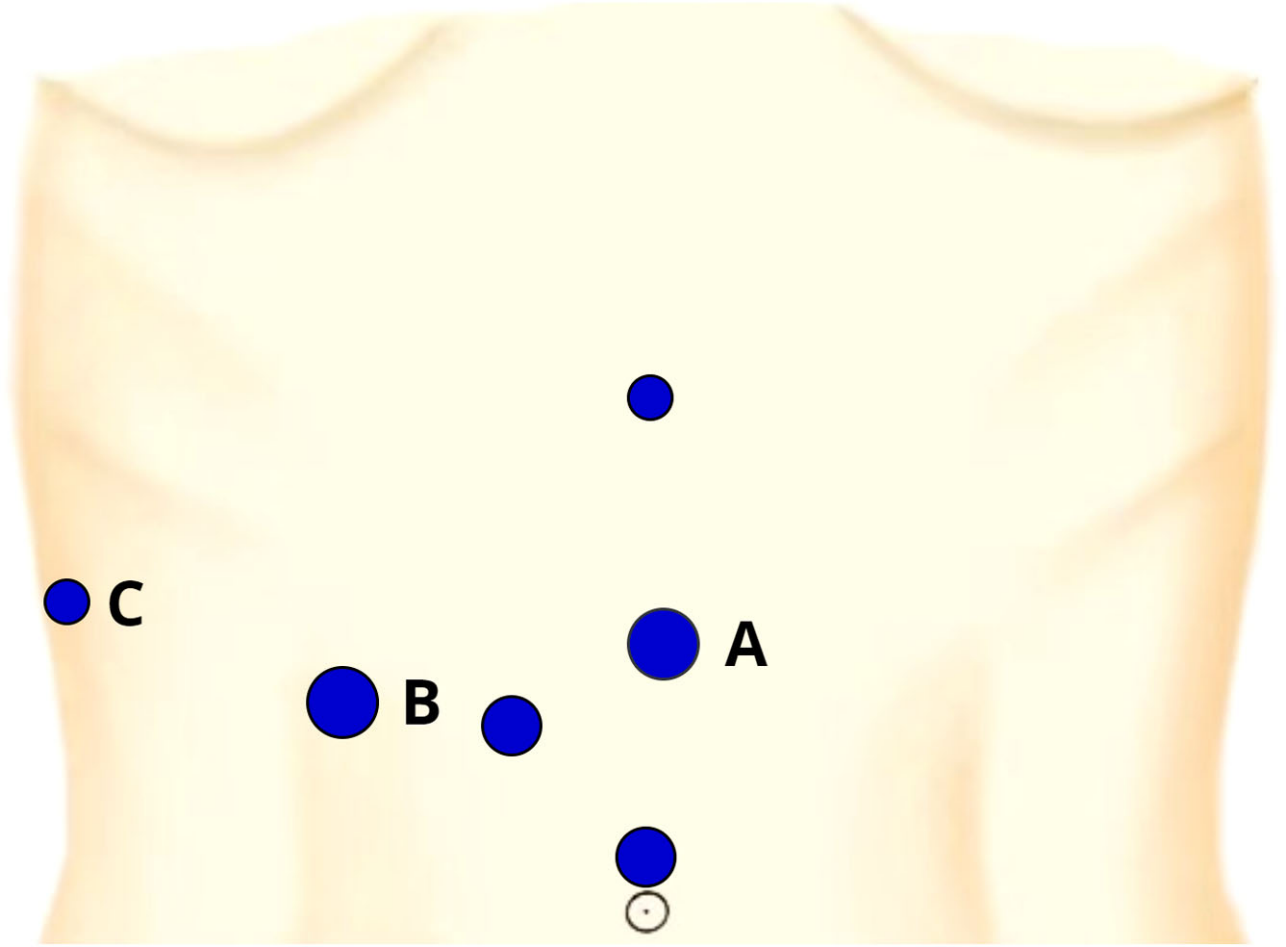
The six-port placements and their entrance for our positive staining. Port **(A)**: a 12-mm trocar located on the left side of the midpoint of xiphoid-navel line, the entrance of the LUS; Port **(B)**: a 12-mm trocar at the junction of 5 cm below the costal margin and the midclavicular line, the entrance of the needle and laparoscopic needle holder; Port **(C)**: a 5-mm trocar located at the junction of the anterior axillary line and the costal margin, the entrance of the laparoscopic noninvasive forceps.

The six-port placements of the operation are illustrated in **Figure 2**. The first assistant stood at the patient’s left side and located the target portal vein by the LUS (port A). The senior surgeon stood at the patient’s right side, set the customized puncture needle into the abdominal cavity (port B), and punctured the needle through the adaptor into the target portal vein by using a laparoscopic needle holder (port B) and noninvasive forceps (port C). Blood was withdrawn to ensure that the needle tip was in the target portal vein, and 5-10 ml (depending on the segmental volume) of 0.025 mg/ml ICG solution was slowly injected into the vessel. After injection, the fluorescence demarcation line was identified in several seconds and delineated through electrocautery under ICG fluorescence imaging.

### Procedural techniques

Before staining, adequate dissection of the liver is necessary in most cases, especially for the dorsal puncture in S7. After positive staining, the liver parenchyma was transected using a harmonic scalpel (Ethicon, NY, USA) using the clamp-crushing technique. Vessels with a diameter of 2 mm or less were directly dissected using a harmonic scalpel, while vessels with a diameter greater than 2 mm were dissected after clipping with a hemostat clamp or stapler. Hemostasis of the cut surface was achieved using a bipolar sealer or monopolar electrocautery. The hepatic blood inflow was controlled by means of intermittent application of Pringle’s maneuver with 15 min of occlusion and 5 min of release to reduce blood loss during liver parenchymal transection. In the transection, fluorescence imaging can be displayed repeatedly to confirm the demarcations.

## Results

### Demographic data and intraoperative and postoperative outcomes

**Figure 3:**
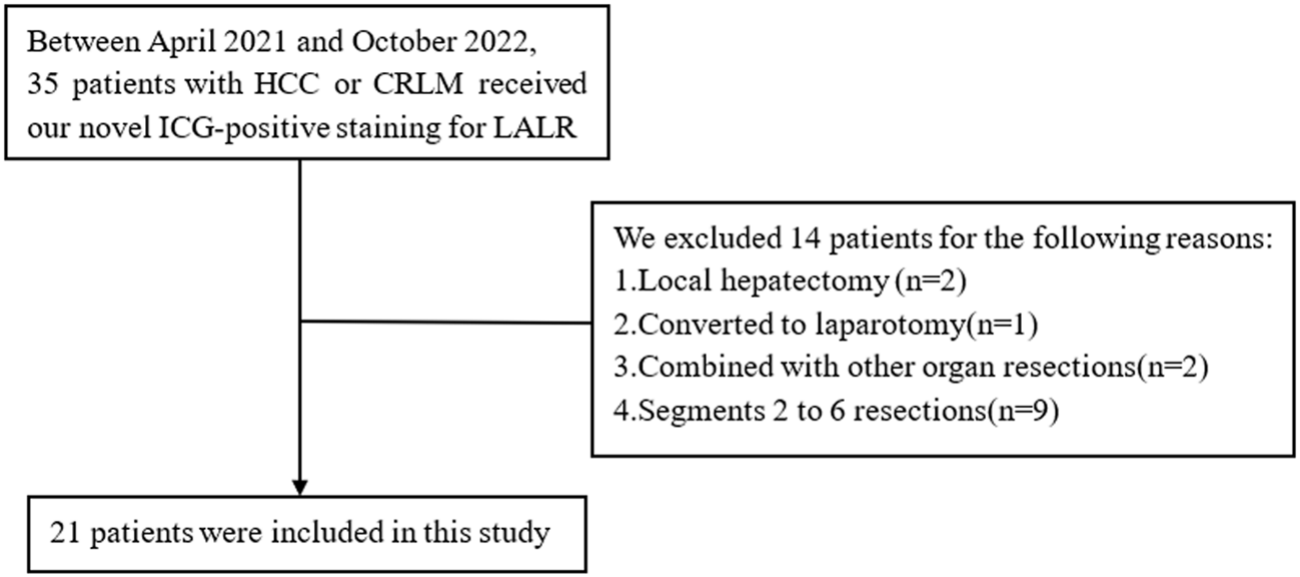
Flow chart of patient inclusion.

In total, 35 patients with HCC or CRLM underwent our novel positive staining for LALR, but 14 patients were excluded, including local hepatectomy(n=2), converted to laparotomy(n=1), combined with other organ resections(n=2), and segments 2 to 6 resections(n=9). Finally, 21 patients underwent LALR of the right superior segments with this novel positive staining method (**Figure 3**). Among the 21 patients, ten underwent S7 hepatectomy, six underwent S8 hepatectomy, two underwent S8v hepatectomy, and three underwent S8d hepatectomy. Fourteen cases were diagnosed with hepatocellular carcinoma and seven cases were diagnosed with colorectal liver metastasis according to the postoperative pathology. The mean age of the 21 patients (15 males and 6 females) was 61 years, ranging from 28 to 85. The average staining time (including LUS location, needle puncture and ICG injection) was 13.0 ± 6.4 min, the operative time was 230.4 ± 71.7 min, the blood loss was 140.5 ± 106.8 ml, R0 resection was 100%, and the postoperative hospital stay was 7.1 ± 2.4 days (**Table 1**).

**Table 1:** The data of 21 patients.

Variables	Frequencies	Range/Percentage
Sex		
male	15	71.4%
female	6	28.6%
Age(year)^a^	60.7 ± 14.9	28-85
Pathology		
HCC	14	66.7%
CRLM	7	33.3%
Tumor size(mm)^a^	33.6 ± 14.9	11-74
HBV/HCV	13	61.9%
Cirrhosis	10	47.6%
Segments of staining		
S7 S8 S8v S8d	10 6 2 3	47.6% 28.6% 9.5% 14.3%
Operative time(min) ^a^	230.4 ± 71.7	120-395
Staining time(min) ^a^	13.0 ± 6.4	5-30
Blood loss(ml) ^a^	140.5 ± 106.8	50-500
Success cases	15	71.4%
Postoperative hospital day(d) ^a^	7.1 ± 2.4	5-16
Severity of complications^b^		
I II III IV V	2 0 2 0 0	9.5% 0 9.5% 0 0

HCC, hepatocellular carcinoma; CRLM, colorectal liver metastasis; HBV/HCV, hepatitis B virus/hepatitis C virus; ^a^ Data are expressed as the mean ± standard deviation (SD); ^b^ Clavien–Dindo classification of surgical complications.

The overall success rate was 71.4%. Six failure cases were classified into three types in our practice as follows: FI (Failure I)- unable to puncture into the target portal vein (n=2), poor location or thin vessel (<3 mm); FII (Failure II)- nontumor-bearing segment staining (n=2), backflow or diffusion. Of note, slight contamination could also be considered successful, as we could distinguish the surgical boundaries according to the intensity of fluorescence in fluorescent mode or grayscale in grayscale-mode; FIII (Failure III)- tumor-bearing segments with incomplete staining (n=2), puncture of the tributary of the target vessel or incomplete puncture of multiple target branches. In FI, one patient underwent S7 hepatectomy, deep puncture and limited needle length led to puncture failure, and in the other patient who underwent S8d hepatectomy, two relatively thin target vessels led to failure. In FII, two patients underwent S8 hepatectomy, ICG back flowed into S5 (segment V) in one patient, and ICG diffused into S7 because of vascular anatomic variation in another patient. In FIII, one patient who underwent S8 hepatectomy underwent puncture of two of multiple (>3) target branches, and the other patient who underwent S7 hepatectomy underwent puncture into the tributary of target P7 (portal vein of segment VII), leading to incomplete staining (**Table 2**). In failure cases, LALR can be completed with negative staining or the method inducing ischemic demarcation (an incomplete LALR with the help of LUS and landmark veins) after inflow clamping of the target territory.

**Table 2:** Staining outcomes and Failure causes.

Segment	Outcome	Failure cause	Failure type	Margin
S7(n=10)	Success(n=8)			standard
	Failure(n=2)	Deep puncture	FI	none
		Puncture into tributary	FIII	smaller
S8(n=6)	Success(n=3)			standard
	Failure(n=3)	Backflow into S5	FII	larger
		Diffuse into S7	FII	larger
		Incomplete puncture	FIII	smaller
S8v(n=2)	Success(n=2)			standard
S8d(n=3)	Success(n=2)			standard
	Failure(n=1)	Thin target vessels	FI	none

There were 4 (19.0%) patients with postoperative complications. Two patients with slight bile leakage recovered with conservative treatment, and two patients with pleural fluids recovered after thoracic tube drainage. No Clavien−Dindo IV or V complications occurred in this study.

### Patients’ procedures using ICG-positive staining

#### Segment VII staining

**Figure 4:**
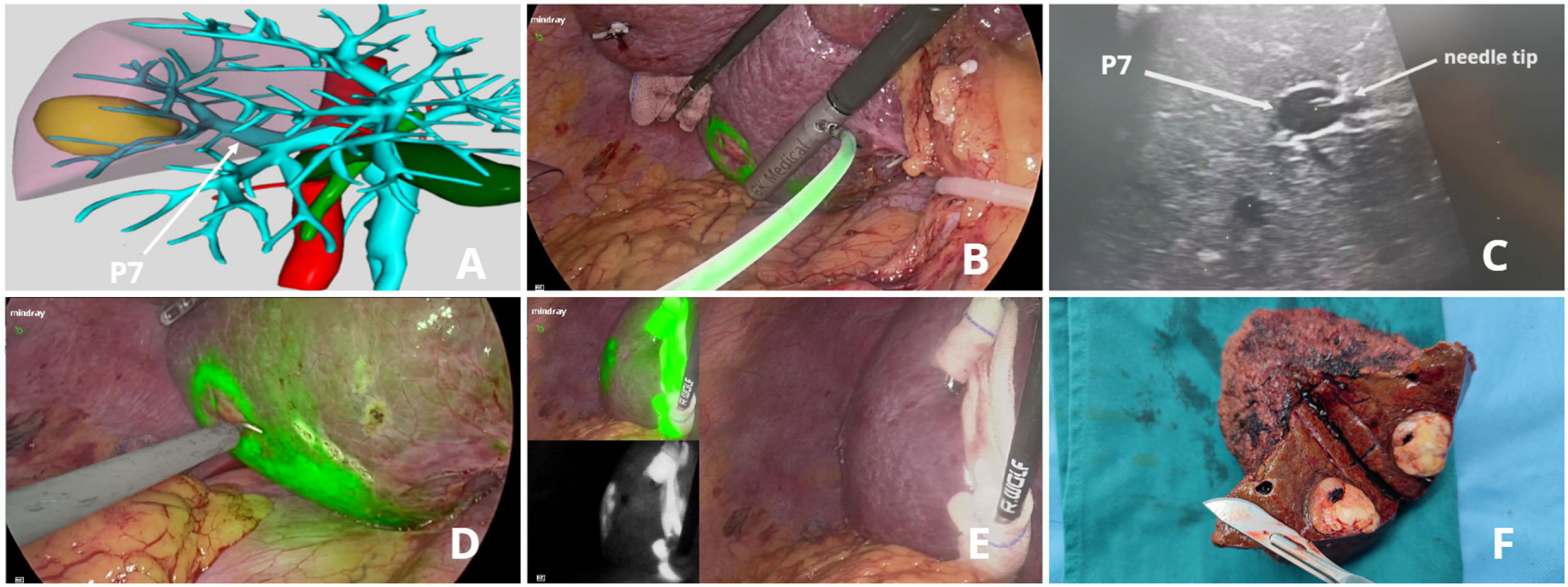
ICG fluorescence-guided S7 segmentectomy. **(A)** preoperative 3D simulation of CT identified the tumor confined to segment 7, located at the tumor-bearing portal branch (P7); **(B)** ICG solution injection from the liver dorsal surface; **(C)** LUS guided puncture to P7: ICG solution was floating in P7 through the needle tip; **(D)** fluorescent delineation of tumor-bearing S7 on the liver dorsal surface; **(E)** liver ventral surface demarcation; **(F)** specimen.

A 48-year-old male patient with a single HCC (33 mm x 24 mm) confined to Segment 7 (Patient 5). We performed this novel method with a customized needle to puncture P7 from the liver dorsal surface and injected 5 ml ICG solution. **Figure 4** (See **Video 1, Supplementary digital materials**)

#### Segment VIII staining

**Figure 5:**
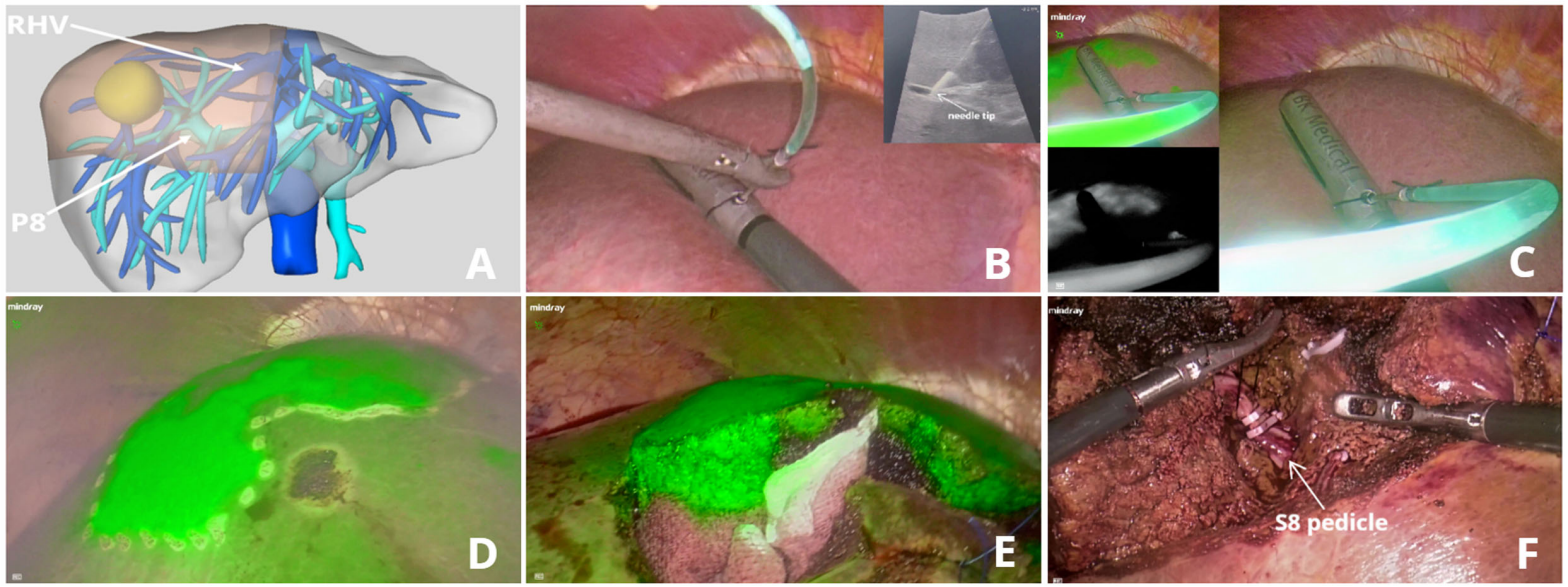
ICG fluorescence-guided S8 segmentectomy. **(A)** Preoperative 3D simulation of CT identified the tumor confined to segment 8, located in the tumor-bearing portal branch and the landmark hepatic veins; **(B)** LUS-guided puncture to the portal branch feeding S8; **(C)** ICG solution injection process under fluorescence imaging; **(D)** fluorescent delineation of tumor-bearing S8; **(E)** intrahepatic parenchymal borderline in the transection; **(F)** S8 pedicle dissection and division.

A 68-year-old female patient with a single HCC (24 mm × 18 mm) confined to segment 8 (Patient 15). P8 (portal vein of segment VIII) was punctured from the liver ventral surface, and 5 ml ICG diluted solution was injected. **Figure 5** (See **Video 2, Supplementary digital materials**)

## Discussion

Laparoscopic anatomical liver resections guided by a demarcation line after portal staining or inflow clamping of the target area have been established as essential methods for curative treatment of hepatocellular carcinoma (HCC) and have subsequently been applied to other malignancies (15). However, located deep-seated and surrounded by the ribs and the diaphragm (16), there is still no standardized approach to right superior segments.

With the emergence of fluorescence imaging techniques, ICG staining is increasingly applied in LALR. However, both conventional positive staining and negative staining of ICG injection are technically demanding and have a poor success rate. Xu et al.’s (9) 36 cases of LALR guided using ICG concluded that segmental staining was successful in 19 procedures (53%, 19/36), including negative staining and positive staining in 52% (14/27) and 56% (5/9), respectively. Diffusion of ICG might be the biggest problem with negative staining, which may lead to a smaller than expected resection area and a narrower surgical margin (17). Lan et al. (17) reported 24 LALR procedures using a positive staining “hepatic pedicle first” approach, representing a 79.17% success rate. However, both negative staining and the “hepatic pedicle first” approach require dissection of the target pedicle, which is technically demanding and time-consuming. Moreover, the pedicles of S7/8 are rather difficult to dissect through the first porta. Similarly, the other approach, in which the Glissonean pedicle supplying the tumor-bearing segment(s) is clamped to induce an ischemic demarcation line on the liver surface, is mainly feasible for Segments 1 to 6 and difficult for the superoposterior segments (6). Indeed, the method inducing ischemic demarcation has other problems, including identification in intrahepatic parenchymal borderlines and undetectable demarcation in some difficult settings. Percutaneous transhepatic puncture with a PTCD (percutaneous transhepatic cholangial drainage) needle is a common method of positive staining in Asian centers. In Takeshi Aoki’s report, two cases of preoperative percutaneous ICG injection failed, representing an 86% (12/14) success rate (14). We have practiced this method and experienced several complications using the procedure. First, to perform this method, surgeons must be skillful at the interpretation of the preoperative 3D simulation, proficient in LUS and comfortable with ultrasound-guided puncture. Second, due to the limitations of the abdominal wall and PTCD needle inflexibility, a precise transhepatic puncture is quite difficult in laparoscopic settings. Surgeons used educated guesses to determine puncture points and experienced manipulation to puncture or adjust the needle. In other words, percutaneous transhepatic puncture cannot be replicated easily. Compared to the method using a PTCD needle, our method guided by the guide line and guide hole is more precise and easier to learn. In addition, our customized needle with a soft injection tube is more flexible to manipulate and adjust in the abdominal cavity.

A new positive staining method using a laser fixed on the LUS probe to irradiate the abdominal wall to determine the puncture point seems more innovative, while the abdominal wall might be a big hurdle. The abdominal wall is quite thick in some patients which might influence the puncture path and slight deviation of the puncture path probably cause puncture difficulties. Compared to this method, our method is not limited by the abdominal wall with simpler workmanship. Another approach was reported by Ueno et al., who used preoperative interventional radiology techniques to stain the targeted arterial branch by performing angiography in a hybrid operation room. This procedure is helpful but may not be popular in centers because of its additional time and cost (18).

Our method of positive staining is new, with a relatively high success rate (71.4%) and short staining time (13.0 ± 6.4 min). To our knowledge, this is the first report describing this method for positive staining in LALR of right superior segments. It seems to be effective and efficient. In addition, this set of puncture needles is user-friendly and cost-effective. Our new device was mainly used for ICG-positive staining in laparoscopic anatomical liver resection of S7 and S8. Besides, it also could be used in LALR of segments 2 to 6, but the total 9 cases of five segments were relatively few. In fact, in our clinical practice, S5, S6(segment VI) and left liver resections were generally performed by negative staining, as it seemed easier and more effective than positive staining. Laparoscopic anatomical liver resection of right superior segments (S7 and S8) is the best indication of our method, and it is also our main aim in this study.

Sufficient preoperative and intraoperative preparations would reduce the failure rates. Considering the complicated intrahepatic anatomical structure and vascular variations, it is indispensable to perform preoperative 3D simulation and study it carefully to determine the target vessel(s) or even the exact point(s) on the vessel(s) to be punctured. Over the last decade, it has been proven safe and effective to use 3D simulation software for pre-hepatectomy assessment, virtual hepatectomy, and measurement of liver volumes in blood flow areas of the portal vein (19). Moreover, it has been reported that 3D reconstruction achieves better outcomes than 2D imaging. Jihua Jiang’s meta-analysis of 16 studies showed that the 3D liver reconstruction group was significantly superior to the conventional surgery group in terms of operation time and intraoperative blood loss (20).

In our practice of this study, S7 staining had a better outcome since P7 is generally a single large branch or has fewer small branches. In the P7 puncture, the needle puncture depth might be > 8 cm from the ventral liver surface. However, deep vessels were difficult to puncture, which meant more parenchymal puncture with a higher risk of injury. Therefore, we could puncture from the dorsal liver surface instead, as Patient 5 presented. S8 often has two main portal branches (P8v and P8d, ventral branch of P8 and dorsal branch of P8) or more, so we can dye its subsegments according to our needs. If we want to dye the complete S8, we must identify which branches to be dyed through preoperative 3D reconstruction to avoid omission. When S8 branches were >3 times and the branch diameters were relatively thin, negative staining was chosen instead. Of note, we had undergone a failure of S8 staining because of backflow. We targeted the puncture site of P8 through LUS. However, the puncture point on the vessel was too close to the proximal portal venous bifurcation, making ICG backflow to S5 apparent and even visible in LUS imaging. Therefore, similar to the conclusion of Pro. Makuuchi, the puncture point of the portal venous branch must be 1 to 2 centimeters distal from the ligating point because the dye regurgitates in the portal vein and flows into the other branches, which should be preserved (5). In addition, a slow injection can avoid backflow to some extent.

There are several limitations to our staining procedure. First, backflow, diffusion and multiple thin target vessel(s) are still tricky challenges when using this method. In addition, the needle and adaptor we devised may be a transitional product. An advanced product could be an integrated needle with LUS. Third, this study was retrospective with a relatively small sample size. A prospective study with a large sample size should be developed in our future study.

## Conclusions

In conclusion, this novel method of positive staining in LALR of right superior segments seems to be effective, efficient and convenient. Further study may involve the integrated design of a puncture needle with LUS including workmanship, materials and specifications. With the refinements of laparoscopic instruments and the improvement of surgeons’ techniques, this method can be applied further.

## Data availability statement

The original contributions presented in the study are included in the article/**Supplementary Material**. Further inquiries can be directed to the corresponding author.

## Ethics statement

The studies involving human participants were reviewed and approved by the Ethical Review Board of the Second Affiliated Hospital of Zhejiang University. The patients/participants provided their written informed consent to participate in this study. Written informed consent was obtained from the individual(s) for the publication of any potentially identifiable images or data included in this article.

## Author contributions

SY and BZ performed the operation on all patients and designed the research. ZJ and XZ wrote the manuscript. GL, ZG, YT, CS, and SX participated in the operation and collected the data. ZJ, BZ, and XZ analyzed and interpreted the data and revised this article. All authors contributed to the article and approved the submitted version.

## Supplementary material

Supplementary Video 1 Positive staining in laparoscopic anatomical S7 segmentectomy.

Supplementary Video 2 Positive staining in laparoscopic anatomical S8 segmentectomy.
